# Practicability for robot-aided measurement of knee stability in-vivo

**DOI:** 10.1186/s12891-015-0826-5

**Published:** 2015-12-03

**Authors:** Andrea Lorenz, Verena Krickl, Ingmar Ipach, Eva-Maria Arlt, Nikolaus Wülker, Ulf G. Leichtle

**Affiliations:** Department of Orthopaedic Surgery, University Hospital Tübingen, Hoppe-Seyler-Straße 3, Tübingen, 72076 Germany

**Keywords:** ACL, Anterior tibial translation, Rotational stability, Robotic-aided, In-vivo measurement

## Abstract

**Background:**

For the analysis of different treatments concerning anterior cruciate ligament (ACL) rupture, objective methods for the quantification of knee stability are needed. Therefore, a new method for in-vivo stability measurement using a robotic testing system should be developed and evaluated.

**Methods:**

A new experimental setting was developed using a KUKA robot and a custom-made chair for the positioning and fixation of the participants. The tibia was connected to the robot via a Vacoped shoe and magnetic buttons, providing adequate safety. Anterior tibial translation and internal tibial rotation were measured on both legs of 40 healthy human subjects at 30°, 60° and 90° of flexion, applying anterior forces of 80 N and internal torques of 4 Nm, respectively.

**Results:**

While the mean differences between the right and left leg measured for anterior tibial translation were within an acceptable range (<1.5 mm), the absolute values were substantially large (38–40.5 mm). For mean internal tibial rotation, between 17.5 and 20° were measured at the different sides and flexion angles, with a maximal difference of 0.75°. High reproducibility of the measurements could be demonstrated for both, anterior tibial translation (ICC(3,1) = 0.97) and internal tibial rotation (ICC(3,1) = 0.94).

**Conclusions:**

Excellent results were achieved for internal tibial rotation, almost reproducing current in-vitro studies, but too large anterior tibial translation was measured due to soft-tissue compression. Therefore, high potential for the analysis of ACL related treatments concerning rotational stability is seen for the proposed method, but further optimization is necessary to enhance this method for the reliable measurement of anterior tibial translation.

## Background

Anterior cruciate ligament (ACL) rupture is one of the most common sport injuries with an annual incidence between 0.15 and 3.76 % for different sports and countries [1]. Although ACL replacement surgery has become a standard procedure in orthopaedic hospitals all over the world, optimal treatment is still discussed controversially [2–4] and a multitude of surgical techniques is commonly in use. Therefore, not only for the indication of ACL replacement but also as evaluation and performance control of the surgical treatment as well as for scientific comparison of these surgical techniques, objective methods for the quantification of knee stability are needed. In the clinical practice, the Lachman and anterior drawer tests are used for testing anterior stability of the knee, while the pivot shift test is applied to check for rotational stability. However, these clinical tests only enable the classification of knee stability into different grades. Neither objective force application nor proper quantification of anterior and rotational stability can be achieved. Therefore, several devices and methods for more accurate stability measurement have been developed.

Considering anterior knee stability, simple mechanical devices, like the Rolimeter [5], electromagnetic [6] or electrogoniometric [7] devices, as well as intraoperatively applied computer navigation systems [8, 9], have been used for the measurement of anterior shift during manual application of the Lachman or drawer tests. More advanced devices regarding controlled force application are the KT-1000 [10, 11], where certain force levels are acoustically indicated, and the KT-2000 [12] with an additional plotter for the attained force/displacement curve. This device was also identified as benchmark for the in-vivo testing of anterior knee stability by a recent review [13], while the Rolimeter was demonstrated to provide comparable results [13–15].

For rotational stability, the comparison of different devices is even more difficult, as different output measures were considered. During manual application of the pivot shift or simple rotation tests, either the resulting tibial rotation angle [8, 9], the coupled anterior tibial translation [16] or the posterior acceleration during the reduction incident [6, 16, 17] were evaluated. For the application of simple rotational torques, the possibility for additional visual control of the force level was realized in more advanced testing devices [18–20]. Branch et al. [21] even introduced a testing device, where the rotational torques could be automatically applied by a system of servo motors. However, for the objective measurement of rotational stability no method has been established as a gold standard yet.

In particular with regard to controlled load application, reliable and objective measurements of anterior and rotational knee stability could only be achieved in-vitro, so far. Most of these in-vitro studies where using robotic systems, evaluating anterior tibial translation during anterior force application (88 N or 134 N) as well as during simulated pivot shift test (10 Nm valgus torque + 4, 5 or 10 Nm internal rotational torque [22–32]. Some studies additionally considered the rotational range [22, 23, 25, 30, 32]. Robotic devices equipped with force/torque sensors are able to apply exactly reproducible loadings while measuring the motion of the tibia in 6° of freedom with high accuracy. However, using in-vitro experiments, no conclusions can be made on the performance after healing or even a long-term period and it is not possible to relate biomechanical parameters to patient satisfaction or quality of life.

Due to the large potential of robot-aided knee stability testing, the aim of the current study was to adapt this major in-vitro technique for the application in-vivo and to analyse practicability for the measurement of rotational and anterior knee stability. Our first hypothesis was that the new method enables safe and secure measurement of knee stability, without clinically relevant danger or pain for the participants. As for in-vivo measurement of rotational stability, there is no real gold standard available, yet, this was our main focus. Thus, our second hypothesis was that our method allows reproducible measurements of rotational knee stability and provides comparable results as recent in-vitro studies. Finally, as anterior stability is crucial concerning ACL treatment, our third hypothesis was that our method also works for anterior stability providing reproducible and comparable results to the Rolimeter, a well established standard method.

## Methods

**Fig. 1:**
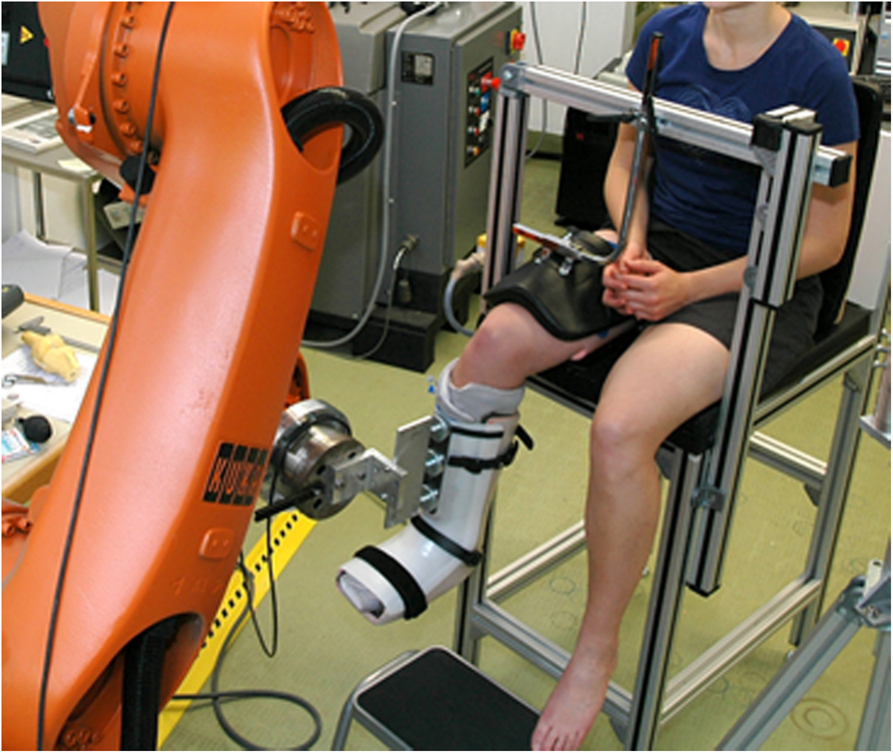
Experimental setting for the robot-aided measurement of knee stability in-vivo. Anterior tibial translation and internal tibial rotation were measured using a KUKA robot while the participants were positioned in a custom-made chair

Anterior stability and rotational range of the knee joint were measured in-vivo using a robotic device. For this purpose, a new experimental setting was developed (Fig. 1) and tested on 40 healthy human subjects. The experiments were approved by the ethical committee of the University of Tübingen (reference number: 228/2008MPG1).

### Experimental setting

**Fig. 2:**
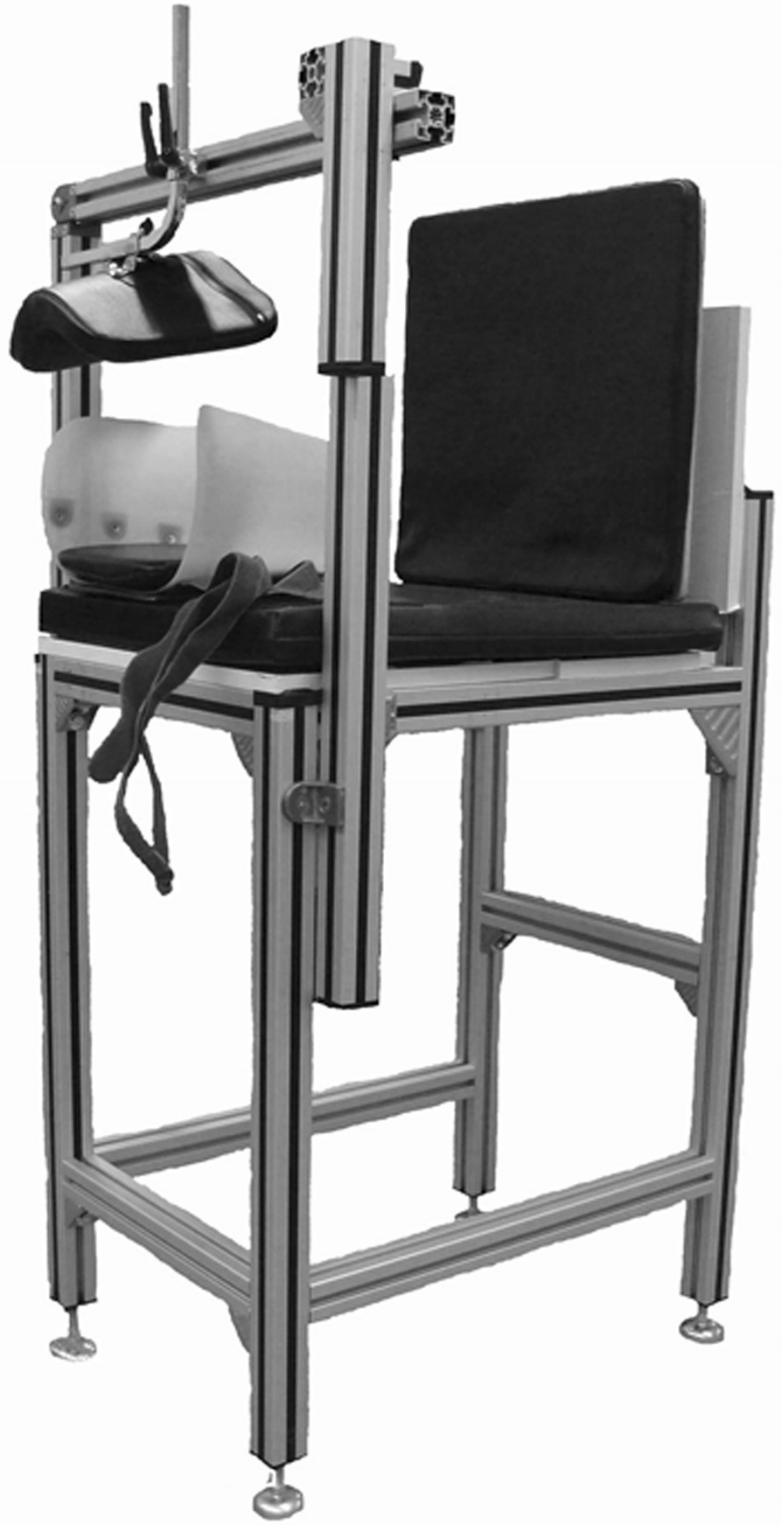
Custom-made chair for the positioning and fixation of the participants. A chair was developed for the positioning of the participants, providing an adjustable backrest and a fixation device for the thigh of the tested leg

**Fig. 3:**
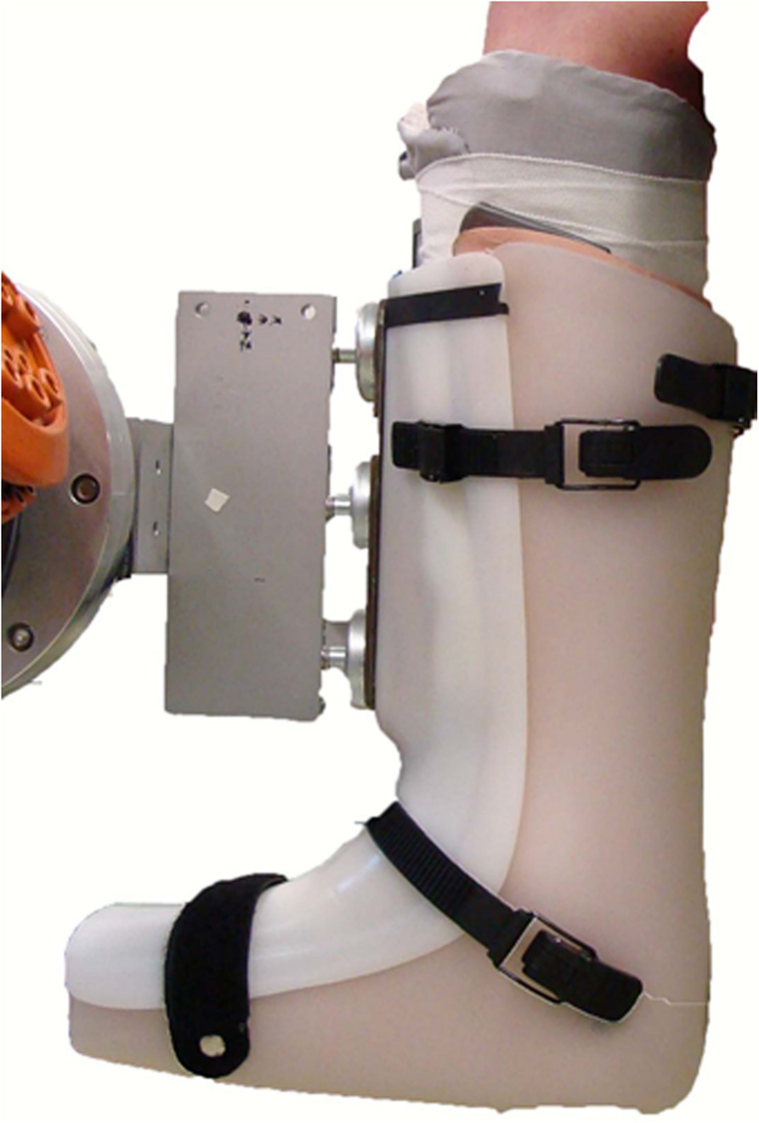
Risk-minimizing coupling mechanism. The robot arm is equipped with 3 magnets, docking to the metal plate inserted at the front of an adopted Vacoped shoe

The experimental setting was based on a KUKA robot (KUKA KR 60–3 robot, Augsburg, Germany; reproducibility: ±0.06 mm) equipped with a universal force/torque sensor (ATI UFS: Theta SI1000-120; resolution: 0.25 N and 0.025 Nm), which had already been applied for knee stability measurements in-vitro [33]. For the measurements, the subjects were positioned in a custom-made chair. The chair provided an adjustable backrest and a fixation device for the thigh of the tested leg, consisting of a plastic shell with 3 Velcro straps and an additional padded piston to minimize vertical motion (Fig. 2). For a secure and safe docking of the robot to the subject, a magnetic coupling mechanism was developed. While the robot arm was equipped with 3 magnetic disks, the subject had to wear an adapted Vacoped shoe (OPED GmbH, Valley / Oberlaindern, Germany) with inserted metal plate at the front, which served as counterpart (Fig. 3). Vacoped shoes have been used for the in-vivo measurement of rotational knee stability before, providing appropriate reproducibility [18, 34].

### Human subjects and clinical examination

Fourty healthy human subjects (mean age 29.13 years, 20 male, 20 female) participated in our study. Subjects had no history of knee injury or surgery, no symptoms of osteoarthritis at both knee joints and were free of pain and other severe health problems. They were informed of the experimental procedure and all possible study risks and signed a consent form approved by the ethical committee of the University of Tübingen.

Prior to testing, the participants had to undergo clinical examination of both knee joints, including the Lachman and drawer tests, the pivot shift test, as well as quantitative measurement of anterior stability using the Rolimeter in 30° and 90° of flexion. The tests were repeated after experimentation to document that there was no injury due to the measurements.

### Experimental procedure

The participants had to sit upright in the examination chair and the backrest was adjusted. A Vacoped shoe was put onto the lower leg of the tested leg, producing vacuum in the inner layer for a better fit and tightly closing the clasps. The upper leg was positioned into the plastic shell, the 3 straps were closed and the piston was adjusted and fixed onto the thigh under slight pressure. For reproducible definition of coordinate systems, the center of the knee was determined with respect to the robot docking point. The robotic coordinate system used for motion tracking and force control was defined with its z-axis in proximodistal direction along the tibia, its y-axis in lateromedial direction perpendicular to the y-axis and approximately through the femoral epicondyles and its x-axis in posterioanterior direction, perpendicular to the y- and z-axes.

Docking of the robot was performed in a relaxed seated position of the subject with the knees flexed at 90°. For this procedure, the magnets fixed to the robot were positioned a few centimeters in front of the metal plate on the Vacoped shoe at the subject’s shank by manual motion control of the robot. Subsequently, the robot was switched to force controlled mode with target values of 0 N and 0 Nm for all forces and torques, respectively. In this mode, the robot arm could be manually moved by an assistant and cautiously docked to the plate via the magnets.

After docking, testing started in 90° of flexion. For the measurement of anterior tibial translation (ATT), an anterior force of 80 N was applied. This force level was chosen similar to the inital KT studies [35–37], where 67 N or 89 N were used and after preliminary testing has shown that it was well tolerated. To prevent pain or damage of any soft tissue at the knee joint, the forces and torques in all remaining directions were controlled to 0 N or 0 Nm, except for the flexion / extension torque, where motion was locked to keep the knee in the respective flexion position. In all other directions, residual motion was possible to eliminate loading. For the measurement of the rotational range, 4 Nm of internal torque were applied, according to similar in-vitro studies [23, 26, 31]. Again, the remaining torque (varus/valgus) and all forces were controlled to 0. In the final position, the force or torque was held for 2 s and then the robot went back to the starting position. Three repetitions of anterior stability as well as rotational range measurements were carried out. During force / torque application, the motion of the robot was measured in 6° of freedom with a sample rate of 12 ms.

After the measurements in 90° of flexion, the robot slowly moved the shank into the next testing position, 60° of flexion, by an automated 30° rotation around the y-axis. During this step, again all forces and torques in the remaining directions were controlled to 0, leading to an individual adaption of the motion path for each participant. Measurements of anterior and rotational stability were repeated in 60° and 30° of flexion. Prior to each measurement, the force sensor was calibrated to adjust for the weight of the subject’s lower leg. Subsequently, the thigh fixation and shoe were changed to the other side and the whole procedure was repeated on the second leg. The sequence of the right and left leg was alternated.

### Safety mechanisms

To minimize risk and guarantee safety of the participants during the whole experimental procedure, several safety mechanisms were implemented. First, the magnetic discs were chosen such that the participant was able to autonomously undock his or her leg from the robot by a simple side turn of the shank or by activation of the hamstring muscles. Each subject was allowed to train undocking prior to the measurements. Preliminary tests without human subjects had demonstrated automatic undocking below 100 N during anterior force application (maximum at 30° of flexion: 99.68 N) and below 9 Nm during internal torque application (maximum at 90° of flexion: 8.97 Nm). Furthermore, the chair was positioned at the border of the robot’s coverage, which made it impossible for the robot to push back the subject. In the robotic software, forces and torques were limited to 250 N and 25 Nm, while the movement was limited to 60 mm and 30° from the starting position. After all, an additional emergency switch was positioned at the right side of the chair.

### Data analysis

Maximal anterior tibial translation and internal tibial rotation were calculated as the mean value over the last two seconds, where the final position was hold. The results of the 3 consecutive measurements were averaged. The force and torque levels reached at the end of each repetition were also evaluated (mean value of last 2 s) and repetitions with anterior forces <75 N and rotational torques <3 Nm were excluded from averaging as these results were attributed to unintended early stops of the measurements. This was the case for 1 of the 3 repetitions in less than 2 % of anterior translation measurements, but in approximately 25 % of tibial rotation measurements. Data of the right and left leg were compared in the different flexion positions and Wilcoxon signed-rank tests were carried out at a significance level of 0.05. Power analysis was performed to significantly distinguish clinically relevant side-to-side differences of 5 mm or 5°.

The coefficient of variation and intra class correlation coefficient (ICC) were calculated as measures of reproducibility. The two-way mixed models ICC(3,k) and ICC(3,1) were used both, because all experiments were and will be carried out by our robot, and it should be determined if the triple repetition of measurements is necessary in future applications.

To check for reliability, the Bland-Altman plot [38] was used, comparing side-to-side differences of anterior translation measured by the robot and the Rolimeter. As no reference measurement was available for tibial rotation, our data was compared the data of a self-conducted in-vitro study and to literature.

## Results

All 40 participants could be examined without causing any pain or detectable damage. Clinical examination after robotic testing delivered no pathological findings just as before. The data of one participant were excluded because measurements were stopped by the safety limits of 60 mm of anterior translation and 30° of internal rotation.

Pivot shift test was negative in all 78 considered knees. For the Lachmann Test (in 30° of flexion), 9 knees were classified between 5 and 10 mm while the remaining 69 were classified below 5 mm. For the anterior drawer test (in 90° of flexion), the classification delivered 8 knees between 5 and 10 mm and 70 knees below 5 mm. Rolimeter testing in 30° of flexion yielded 6.06 ± 1.95 mm (mean ± standard deviation) at the right knees and 6.52 ± 2.13 mm at the left knees, while it yielded 4.17 ± 1.60 mm at the right knees and 3.92 ± 1.40 mm at the left knees in 90° of flexion.

The robotic evaluation of rotational knee stability yielded mean values between 17.5 and 20° (Table 1). At 30° of flexion, the largest rotation was measured. The difference between the right and left knee was largest at 30° of flexion as well and remained below 1° in all flexion angles. The Wilcoxon test revealed a significant difference at 90° of flexion (*p* = 0.03, Table 1). However, this difference (−0.74°) was not clinically relevant. The statistical power to distinguish clinically relevant differences was >0.8 for all cases, indicating that enough specimens were measured. For all measured conditions, the coefficient of variation was below 5 %. Almost perfect ICC (ICC(3,k) = 0.98 and ICC(3,1) = 0.94) was also reached for tibial rotation.

**Table 1:** Results for the robotic measurement of internal tibial rotation

Flexion angle	Internal tibial rotation			
	Right leg	Left leg	Difference	*p*-value
30°	19.71 ± 4.95	18.96 ± 5.14	0.75 ± 3.47	0.22
60°	18.67 ± 4.64	18.22 ± 4.59	0.45 ± 3.43	0.30
90°	17.80 ± 4.07	18.55 ± 4.08	−0.74 ± 3.24	0.03*

Mean values ± standard deviations are reported at 30°, 60° and 90° of flexion, comparing the right and left leg (difference = right-left). The *p*-value is given for the Wilcoxon signed-rank test. The asterisk * is indicating a significant difference

**Fig. 4:**
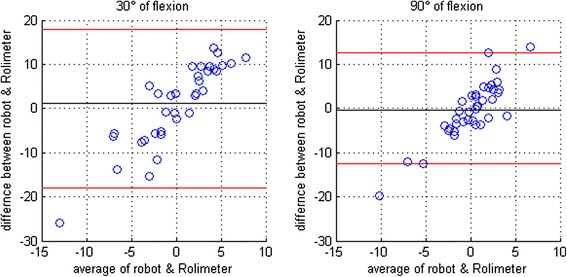
Bland-Altman plots for anterior translation comparing robot and Rolimeter. Right/Left (r/l) differences of anterior translation measured with the robot and the Rolimeter are compared for 30° and 90° of flexion using the Bland-Altman plot

Regarding anterior knee stability, robotic measurement revealed substantially larger tibial translation than the Rolimeter measurement with mean values between 38 and 40.5 mm for the different sides and flexion angles (Table 2). Anterior tibial translation was largest in 60° of flexion, followed by 30° of flexion and 90° of flexion. The differences between the right and left knee were not significant and remained below 1.5 mm for all flexion angles. However, the Bland-Altman plot (Fig. 4) revealed a large variance of these side-to side differences, especially for the 30° measurement, as well as increasing error with increasing translation. The coefficient of variation calculated for the three consecutive measurements was at a maximum of 2.5 % for both legs and all flexion angles. Excellent reproducibility of anterior tibial translation measurement could be further demonstrated using the intraclass correlation coefficient (ICC(3,k) > 0.99 and ICC(3,1) = 0.97).

**Table 2:** Results for the robotic measurement of anterior tibial translation

Flexion angle	Anterior tibial translation			
	Right leg	Left leg	Difference	*p*-value
30°	39.61 ± 8.73	38.67 ± 7.28	0.83 ± 8.66	0.34
60°	40.23 ± 5.99	38.77 ± 6.39	1.47 ± 6.73	0.25
90°	38.05 ± 5.95	38.11 ± 5.68	−0.06 ± 6.09	0.65

Mean values ± standard deviations are reported at 30°, 60° and 90° of flexion, comparing the right and left leg (difference = right-left). The *p*-value is given for the Wilcoxon signed-rank test

## Discussion

A new experimental setting for the in-vivo measurement of anterior and rotational knee stability was developed. It was the first time that this successful in-vitro method [23, 24, 26–32] was applied to living subjects. In our study, 40 healthy human subjects were tested without critical incident, damage or pain for any participant and without pathological findings in the subsequent clinical examination, demonstrating that robotic knee stability measurement is basically possible in-vivo and confirming our first hypothesis.

Considering tibial rotation, no clinical data of the same individuals are available for comparison. However, a multitude of robotic in-vitro studies exists, where the tibia and femur were rigidly fixed to either the ground or the robot. Therefore, these measurements can actually be considered as the gold standard for rotational testing. Amongst others, we conducted an in-vitro study ourselves, using 10 human knee specimens and the same technical parameters [39]. In this study, we measured on average 18.59° and 17.91° of internal tibial rotation at 90° and 60° of flexion, respectively. Comparing these results to the current study, differences of less than 0.6° occur. As 30° of flexion was not measured in our own study, our findings were compared to other in-vitro studies [23, 30, 32, 40]. Despite large variations of the applied torques (either 4, 5 or 10 Nm of internal torque, mostly combined with 10 Nm of valgus torque), all these studies reported similar values at the 30° position, between 18 and 22.5° of internal rotation, fitting well to our result (19.34°). Interestingly, for higher flexion angles the internal rotation angle measured in the different studies either increased, decreased or alternated, leading to large differences in 90° of flexion (between 7.7 and 26.0°).

The variation found in different in-vivo studies was similarly high. In 30° of flexion, Branch et al. [21] measured 18.85° of internal rotation (averaging male and female), applying 5.65 Nm with an automated measurement device, which is in good accordance with our results. In contrast, Shultz et al. [19] measured only 9.55° of internal rotation with a similar device but manual force application of 5 Nm. With 1.53° (control group) and 1.9° both studies measured a slightly larger side-to-side difference than in our study (0.75). Lorbach et al. [18] was using the same testing shoe (Vacoped, Oped), resulting in an internal rotation of 23.7° and a side-to-side difference of 0.7° for 5 Nm of manual torque application. These large differences measured for internal rotation in different studies are mainly attributed the different experimental settings, including the remaining motion of the hip and ankle joint, variations in the manually applied loading or soft tissue artefacts during measurement. In addition, different definitions of coordinate systems and in particular of the 0° position could have had a large influence, also concerning in-vitro studies. With regard to these large deviations in different in-vivo and in-vitro studies, the high consistency (differences <0.6°) between our in-vitro and in-vivo results at 60° and 90° of flexion using the same robot, testing parameters and 0° position, but different femoral and tibial fixation is considered as excellent, confirming our second hypothesis that our setting is appropriate for the measurement of rotational knee stability.

**Fig. 5:**
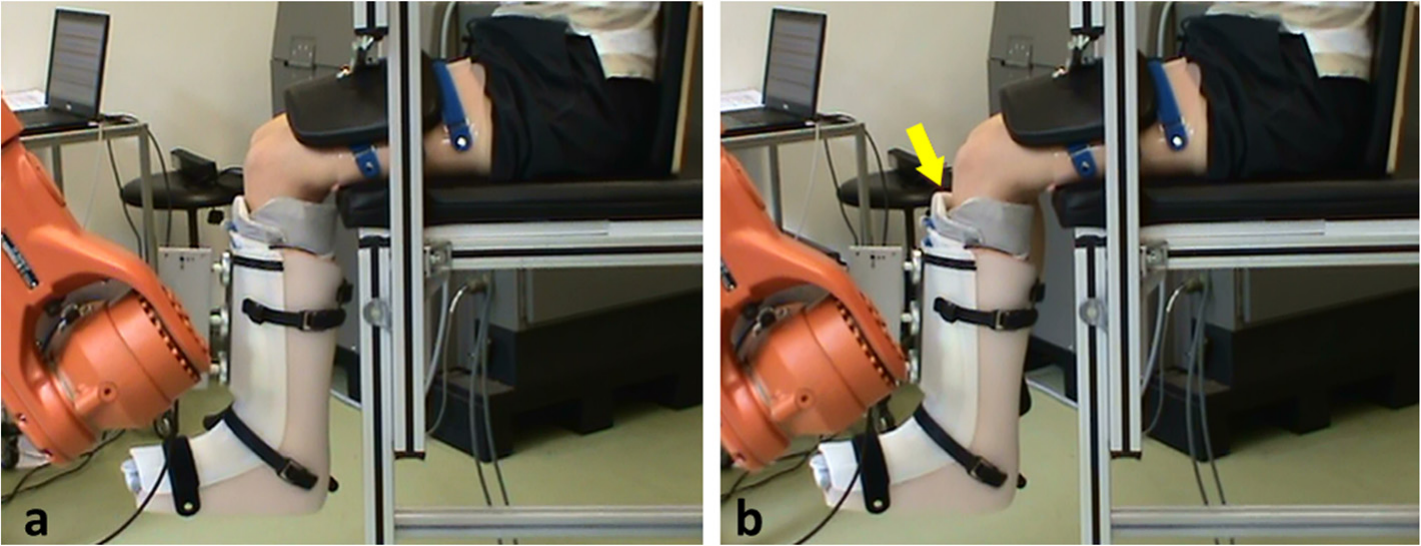
Subject during anterior tibial force application at 90° of flexion. The starting position (**a**) is compared to the position of maximal force (**b**), illustrating the large clearance in the Vacoped shoe (yellow arrow)

However, concerning robotic measurement of anterior knee stability, our experiments also revealed some weaknesses of the proposed method. While the mean difference between the right and left knee measured by the robot (0.83 mm in 30° and 0.06 mm in 90° of flexion) was comparable to the Rolimeter measurement (0.46 mm in 30° and 0.25 mm in 90° of flexion), the actual displacement measured by the robot was on average more than 6 times higher than in the Rolimeter measurement. Mean anterior translation of up to 40 mm was measured using the robot. These extraordinary high values can be attributed to two major reasons. First, due to the compression of the lower leg muscles, large clearance in the Vacoped shoe emerged during anterior force application (Fig. 5). Second, the thigh fixation used in this experimental setting could not totally prevent motion of the femur, which is again primarily attributed to the soft tissue.

These two problems can basically be traced back to a general problem of the robot. As the robot only has one robot arm, force application and motion tracking can only be performed at one single location. The fact that the measurement of tibial translation has to occur at the same location as force application becomes a problem because the robot cannot be attached directly to the tibia during in-vivo experiments. Thus, force application – and therefore motion tracking – has to take place at the backside of the lower leg, where the large shank muscles are located and the clearance due to soft-tissue compression is high. In addition, relative motion of the tibia and femur should be measured, but the robot can only measure motion at one single location, which is the tibia in our experiments. Therefore, the second part – the femur – has to be rigidly fixed to the ground or chair in this case. This is again extremely difficult in-vivo, due to soft-tissue compression and motion.

Although the mean differences between the right and left leg are within an acceptable range during the measurement of anterior tibial translation (<1.5 mm), the absolute values are far too high, just as the standard deviations. These large absolute values are introducing an unwanted source of error even if only the leg difference is considered as interesting. This is illustrated well by the Bland-Altman plots, which shows large variance of the differences and a good correlation between errors and absolute values. Therefore, the third hypothesis that the current setting is also appropriate for the measurement of anterior knee stability has to be rejected. However, facing these problems, we elaborated some suggestions for improvement. First, the fixation of the femur should rather be located at the patella than the upper leg, comparable to measurements with the Rolimeter [5] or the device developed by Branch et al. [21], as at the patella there is almost no soft-tissue that can cause artefacts. Second, the Vacoped shoe should be replaced by some kind of narrow bracket around the upper part of the lower leg, where the shank muscles are tighter and less voluminous. Although this cannot totally prevent soft tissue compression, it is supposed to reduce the artefacts. However, as long as motion tracking is performed with the robot at the same location as force application at the back of the lower leg, there will always remain some errors in the measurement of anterior tibial translation.

With regard to reproducibility, excellent results have been achieved for both, ICC(3,k) and ICC(3,1). From this viewpoint, the triple repetition of each measurement does not need to be carried out necessarily during future application. However, especially during the measurement of tibial rotation, an unexpectedly high number of unintended, early stops occurred. These stops are mainly attributed to the motion of the participants during testing. In particular, forward or sideward bending of the upper body is assumed to have a high impact, as this leads to a pull at the hamstrings, increasing the force measured by the robot. Therefore, additional bracing of the subject’s upper body to the backrest is considered as possible improvement for future measurements. Alternatively, supine positioning of the participants is suggested. This would restrict motion of the subjects even more and additionally facilitate the development of a new patellar fixation as described above. Furthermore, a supine position would probably support relaxation of the patients and decrease muscle stimulus, which might additionally increase the accuracy of our measurements.

In addition to ICC, the coefficient of variation also delivered acceptable reproducibility. The values of both coefficients are worse for tibial rotation which is attributed to the smaller mean values while the standard deviation is similar. Furthermore, as the target value for the tibial rotation torque is only 4 Nm, the signal to noise ratio is worse and measurement noise and outliers in the torque signal have a higher impact on the termination of the tests.

A limitation of the study might be the fact, that only healthy subjects were examined. To guarantee reliability and validity of our experimental setting, the examination of subjects with deficient ACL would be preferable. However, for reasons of safety, the practicability of safe, risk-minimizing measurements should be demonstrated on healthy participants, first, which is a usual approach [18–20]. Another limitation could be the low force level. While most in-vitro studies [23, 26, 28–31], but also in-vivo instruments like the KT 1000 [41–43] are applying up to 134 N, we only used 80 N in our study. This is a result of risk minimization again. However, there also exist in-vitro studies applying 88 N [22, 25] and the initial KT studies were using force levels of 67 N and 89 N [35–37]. In preliminary tests with our laboratory staff, we recognized slight irritations of the knee joint after measurements with 100 N and 5 Nm. Although this might have been the consequence of repeated testing, safety had major priority. Therefore, we constrained the anterior tibial force to 80 N and the internal tibial torque to 4 Nm. Furthermore, soft-tissue compression was identified as major problem during the measurement of anterior tibial translation, which could limit the interpretation of data collected with our apparatus. However, detecting such problems was actually one of the main aims of this practicability study. The problem has been extensively analyzed and suggestions for improvement have been made which should be worked on in future studies.

## Conclusions

Robot-aided in-vivo measurements of anterior and rotational knee stability were performed for the first time. The experiments could be carried out safely and without health risk for all 40 participants, demonstrating high reproducibility for both, anterior tibial translation and internal tibial rotation measurements.

For rotational stability, excellent results were achieved, almost reproducing the results of current in-vitro studies. However, considering anterior knee stability, too large anterior tibial translation was measured due to insufficiencies in robot docking and the fixation of the upper leg, leading to large soft-tissue artefacts and increased variance of the side-to-side differences. Taking into account these enhancements, robot-aided in-vivo measurement of anterior and rotational knee stability is considered as a promising method for the analysis of ACL related surgery techniques and treatments.

Therefore, a safe and appropriate method for the measurement of rotational knee stability has been presented, but further optimization and investigation is necessary to enhance this innovative method for the reliable measurement of anterior tibial translation.
